# Physicians’ perceptions and awareness of adverse effects of proton pump inhibitors and impact on prescribing patterns

**DOI:** 10.3389/fphar.2024.1383698

**Published:** 2024-06-26

**Authors:** Abdelmoneim Awad, Abdulaziz Al-Tunaib, Sarah Al-Saraf

**Affiliations:** ^1^ Department of Pharmacy Practice, College of Pharmacy, Kuwait University, Kuwait City, Kuwait; ^2^ Drug Inspection Administration, Ministry of Health, Kuwait City, Kuwait

**Keywords:** proton pump inhibitors, adverse effects, perceptions, awareness, beliefs, prescribing practices, physicians, Kuwait

## Abstract

**Background:** Heightened scrutiny surrounds the inappropriate use of proton pump inhibitors (PPIs) due to concerns regarding potential serious adverse effects (AEs). Understanding the impact of these AEs on real-world practice is crucial. This study aimed to assess physicians’ perceptions, experiences, awareness, and beliefs regarding published data on potential AEs associated with PPIs. Additionally, it sought to determine alterations in PPI prescribing patterns resulting from these AEs, explore attitudes towards PPI use, and ascertain recommendations for PPI use in clinical scenarios with varying levels of risk for upper gastrointestinal bleeding (UGIB).

**Method:** A quantitative, cross-sectional study utilized a self-administered questionnaire, inviting 282 physicians from 55 primary healthcare centers and 334 internal medicine physicians from seven governmental hospitals to participate.

**Results:** With a response rate of 87.8% (541/616), 74% (95% CI: 70.2–77.7) of respondents were somewhat or very familiar with published data on PPI AEs. Among the familiar, 69.5% (CI: 65.2–73.5) had somewhat or very much changed their PPI prescribing patterns. General concerns about AEs when prescribing PPIs were reported by 62% (CI: 56.7–65.1). Respondents displayed awareness of a median (IQR) of 15 (9) different AEs associated with long-term PPI use, including osteoporosis or osteopenia (90.2%), hypomagnesemia (81.5%), vitamin B12 deficiency (80.6%), and bone fracture (80.0%). Respondents believed that PPIs elevate the risk for a median (IQR) of 7 (6) different AEs, with osteoporosis or osteopenia (81.8%) being the most common, followed by hypomagnesemia (67.1%), and vitamin B12 deficiency (62.3%). The most common strategies for PPI de-escalation were PPI discontinuation (61%) and using PPI on-demand/as-needed (57.9%). The majority (87.4%) agreed or strongly agreed that PPI overuse is prevalent in Kuwait and 78.2% emphasized the necessity for large-scale education on rational PPI use for medical staff and the public. In the UGIB prevention scenarios, 43.6% recommended appropriately the PPI discontinuation in the minimal-risk scenario, while 56% recommended appropriately the PPI continuation in the high-risk scenario. Associations and comparative analyses revealed predictors influencing physicians’ practices and attitudes toward PPI usage.

**Conclusion:** These findings lay the foundation for future research and targeted interventions aimed at optimizing PPI prescribing practices and ensuring patient safety.

## Background

Proton pump inhibitors (PPIs) have been demonstrated as the most efficient medications presently available for suppressing gastric acid secretion by parietal cells in the stomach (Grube and May, 2007; Savarino et al., 2017; Herszényi et al., 2020). PPIs constitute one of the most extensively prescribed medications worldwide (Forgacs and Loganayagam, 2008; Savarino et al., 2017; Savarino et al., 2018). The use of PPIs continues to grow annually in both Western and Eastern countries, posing significant challenges for regulatory authorities due to the escalating costs of therapy and the potential risks to patients. Misuse of PPIs is prevalent in inpatient and primary care settings, primarily driven by their prescribing for preventing gastro-duodenal ulcers in patients without risk factors, prophylaxis against stress ulcers in non-intensive care units, standalone steroid therapy, antiplatelet or anticoagulant treatment in patients without a risk of gastric injury, and the overtreatment of functional dyspepsia (Savarino et al., 2018).

Several studies reported the inappropriate prescribing of PPIs at higher doses, prolonged durations than required, and without a clear indication (Atkins and Sekar, 2013; Chia et al., 2014; Metaxas and Bain, 2015; Alqudha et al., 2016; Liu et al., 2020; Rababa and Rababa’h, 2021). In Kuwait, two studies conducted among geriatric patients in primary and secondary healthcare settings highlighted that PPIs were prescribed without any evidence-based clinical indication (Awad and Hanna, 2019; Awad et al., 2022). There is rising attention on inappropriate PPI usage due to concerns about a wide range of potential AEs associated with prolonged PPI treatment, in certain instances, prompting warnings from pharmacovigilance agencies (Castellana et al., 2021; Yibirin et al., 2021). Nevertheless, the clinical relevance of numerous studies remains uncertain as there is a lack of substantial data supporting a definitive causal link in most instances, which might primarily stem from confounding variables (Laine and Nagar, 2016; Savarino et al., 2016; Schoenfeld and Grady, 2016; Freedberg et al., 2017; Vaezi et al., 2017).

The existing clinical evidence concerning the majority of AEs is either weak or contradictory, often limited by retrospective designs and other methodological issues such as selection biases and confounding factors (Savarino et al., 2016). Despite the presence of a plausible underlying biological mechanism, a definite association with PPI use remains elusive (Castellana et al., 2021). In 2019, a large placebo-controlled randomized trial revealed that the utilization of pantoprazole over 3 years did not demonstrate any AEs, except for an increased risk of enteric infections. These results suggest that the use of PPI therapy for a few years carries minimal detectable risk, while potential risks from longer-term usage could not be assessed (Moayyedi et al., 2019).

Epidemiological studies have linked PPIs with pathology in nearly every organ system (Vaezi et al., 2017; Castellana et al., 2021; Yibirin et al., 2021)*,* including acute interstitial nephritis (Sierra et al., 2007), chronic kidney disease (Lazarus et al., 2016), *Clostridium difficile* infections (Leonard et al., 2007; Janarthanan et al., 2012), Salmonella and Campylobacter infections (Leonard et al., 2007), community-acquired pneumonia (Lambert et al., 2015), dementia (Tai et al., 2017), hepatic encephalopathy (Tsai et al., 2017), hepatocellular carcinoma (Song et al., 2020), gastric cancer (Tran-Duy et al., 2016), osteoporosis and bone fractures (Zhou et al., 2016; Liu et al., 2019), cardiovascular events including stroke, myocardial infarction, cardiovascular death, and major adverse cardiovascular events (Casula et al., 2018; Li et al., 2019), death due to cardiovascular disease, chronic kidney disease, and upper gastrointestinal cancer (Xie et al., 2019), vitamin B12, and calcium, magnesium and iron deficiency (Lam et al., 2013; Eusebi et al., 2017; Srinutta et al., 2019; Castellana et al., 2021).

Given the evolving nature of the evidence, there remains a global gap in understanding the healthcare providers’ current perspectives on AEs associated with the long-term use of PPIs. It remains unclear whether they are adjusting their prescribing and deprescribing practices accordingly in response to this evolving evidence base. A limited body of literature globally has addressed healthcare professionals’ perceptions concerning the AEs associated with prolonged PPI utilization and their influence on PPI prescribing practices. Notably, two studies conducted in the United States of America (USA) aimed to enhance understanding of physicians’ contemporary perspectives on PPI-related AEs, their implications for prescribing behaviors, and the perceived efficacy of PPIs in preventing upper gastrointestinal bleeding (UGIB) (Kurlander et al., 2018; Kurlander et al., 2020). Likewise, studies conducted in China and Saudi Arabia involving healthcare practitioners, including physicians, nurses, and pharmacists, aimed to assess their knowledge, attitudes, and behaviors regarding PPI usage. The survey instrument utilized in the Chinese study was adapted with minor adjustments and applied for data collection in the Saudi Arabian study. Among the knowledge questions, only one addressed PPI AEs: “Do you think long-term use of PPI may cause adverse reactions such as osteoporosis, pneumonia, etc.?” (Luo et al., 2019; Asdaq et al., 2021). Furthermore, a study conducted in Syria sought to evaluate physicians’ perceptions of PPIs and their efficacy in UGIB prophylaxis (Swed et al., 2023). However, to date, no published studies within the Middle East and North Africa (MENA) region, including Kuwait, have comprehensively examined physicians’ current awareness and beliefs regarding the published scientific data on eighteen specific PPI-related AEs. Consequently, the present study was designed to assess physicians’ perceptions, experiences, awareness, and beliefs regarding published data on potential AEs associated with long-term PPI use. Additionally, it sought to determine any alterations in PPI prescribing patterns attributable to these AEs, explore attitudes toward PPI use, and ascertain recommendations for PPI use in clinical scenarios with varying levels of UGIB risk. A secondary objective of the study was to identify predictors for the following outcome variables within the study population: familiarity with published scientific data on possible PPI AEs, the extent of change in PPI prescribing patterns, the extent of general concern about PPI AEs, PPI de-escalation strategies, and the appropriate management of the patient scenarios with different levels of risk for UGIB.

## Methods

### Study area

The present study was conducted in Kuwait, situated in the Middle East, covering an area of 17,820 square kilometers and housing a total population of 4,329,989 individuals (World Population Review, 2023). Kuwait’s healthcare system amalgamates public and private sectors, with the former serving as the primary service provider. The public healthcare framework is organized into three primary levels: primary, secondary, and tertiary. Primary healthcare facilities commonly serve as the initial point of contact for patients, offering a spectrum of services, including clinics dedicated to chronic diseases, preventive care, dental services, and maternity care. Secondary healthcare facilities encompass seven hospitals, providing more advanced services through outpatient clinics and a round-the-clock emergency service. Tertiary healthcare facilities specialize in specific medical domains such as cardiac care, speech and swallowing therapy, transplant services, cancer treatment, and dermatology (Al-Taweel and Awad, 2021)*.* Healthcare professionals, including physicians and pharmacists, employed in Kuwait’s healthcare facilities exhibit a varied spectrum of educational backgrounds. Their training and education originate from multiple sources, encompassing institutions within Kuwait, the MENA region, and countries such as the USA, Canada, the United Kingdom, and India.

### Study design and population

A descriptive and cross-sectional study was conducted among internal medicine physicians operating in seven governmental hospitals spread across six governorates, as well as primary care physicians stationed in 55 primary healthcare centers distributed among the same governorates. Data collection took place between February and September 2023. Ethical approval for this study was granted by the Medical Research Ethics Committee, Ministry of Health, under Ethics No: 2022/150.

### Sample size and sampling strategy

The determination of the sample size was based on assuming a 50% response proportion to the main questions due to the absence of prior similar studies in Kuwait. Utilizing the Raosoft sample size calculator (Raosoft, 2004), considering a 5% margin of error and a 95% confidence interval, the sample size was computed for two groups: 1,000 internal medicine physicians and 600 primary care physicians across seven governmental hospitals and 105 primary healthcare centers The minimum calculated sample size was 278 for internal medicine physicians and 235 for primary care physicians. To accommodate an 80% response rate, an intended sample size of 334 internal medicine physicians (secondary care physicians) and 282 primary care physicians should be approached for inclusion in the study. Using stratified and systematic random sampling according to the methodology delineated by the World Health Organization (WHO, 1993), 55 primary healthcare centers were selected from a total of 105 across six governorates. The stratifying variables included geographic location (governorate) and the number of healthcare centers in each governorate. To ensure proportional representation from each of the six governorates, healthcare centers were stratified accordingly. At the time of the study, 105 centers were operating within the six governorates. The number of healthcare centers selected from each governorate was proportional to its total number of centers. Systematic random sampling was then applied within each stratum. A list of healthcare centers in each stratum was compiled, and centers were selected at regular intervals (r) from a randomly chosen starting point. The interval (r) was determined by dividing the total number of centers in the stratum by the number of centers to be sampled. This method ensured that each center had an equal chance of being selected while maintaining proportional representation across the governorates.

### Study questionnaire

The study questionnaire comprised four distinct domains. Three of these domains were adapted from validated questionnaires that were used in the USA (Kurlander et al., 2018; Kurlander et al., 2020). The remaining domain, focusing on attitudes, was adapted from studies conducted in China and Saudi Arabia (Luo et al., 2019; Asdaq et al., 2021). The first domain encompassed questions on demographic, professional, and practice characteristics, familiarity with guidelines concerning the appropriate PPIs use for UGIB prevention, and the availability of decision support systems such as guidelines or professional recommendations aiding in the appropriate PPI continuation or discontinuation. Questions about professional and practice characteristics were specifically tailored to suit the characteristics of the local study population, ensuring their relevance and applicability. The second domain comprised eight questions. These questions delved into aspects such as familiarity with and concern about potential AEs associated with PPIs. Additionally, it explored the frequency with which participants discuss the risks of PPI-related AEs with patients before prescribing, how frequently patients express concerns regarding these risks. Also, it assessed awareness and beliefs regarding the association of PPIs with various AEs. Respondents were asked to indicate their awareness and beliefs about the association of PPIs with 18 different AEs related to long-term use. The specific questions were: “Are you aware that long-term use of PPIs is associated with the following adverse events? (Please select all that apply)” and “Do you believe that long-term use of PPIs increases the risk of the following AEs? (Please select all that apply)”. Within this domain, six specific AEs (Salmonella and Campylobacter infections, hepatic encephalopathy, hepatocellular carcinoma, calcium deficiency, iron deficiency, and hypomagnesemia) were introduced in addition to the existing 12 specific AEs already incorporated in the original validated questionnaire (Kurlander et al., 2020). Also, respondents were asked which of the potential AEs they worry most about when prescribing PPIs. Finally, the domain concluded with a question addressing the frequency of utilization of six different strategies by participants in altering their prescribing practices due to concerns regarding PPI-related AEs. The third domain encompassed eight items to assess the attitudes of the respondents toward PPIs. Meanwhile, the fourth domain presented four clinical scenarios concerning a 70-year-old woman who uses omeprazole 20 mg daily, recently diagnosed with osteopenia, thereby increasing her susceptibility to bone fracture (Zhou et al., 2016)*.* The scenarios presented different levels of the patient’s risk for UGIB, categorized as 1) Minimal risk: history of gastroesophageal reflux disease (GERD), 2) Low risk: use of low-dose aspirin, 3) Moderate risk: concurrent use of low-dose aspirin and warfarin, and 4) High risk: previous peptic ulcer disease and low-dose aspirin usage. For each scenario, respondents were asked to indicate their management approach for the PPI prescription, choosing from three options: continue omeprazole, stop omeprazole, or stop omeprazole, and also start an H_2_-blocker. After the high-risk scenario, respondents were asked about their beliefs regarding the effectiveness of omeprazole in decreasing the risk of UGIB in the patient. In the low-, moderate-, and high-risk scenarios for UGIB, the estimated annual UGIB risks stand at 0.5%, 1.5%, and 2.7% per year, respectively, in the absence of PPI usage (García Rodríguez et al., 2011; Lanas et al., 2013). Appropriate recommendations lean towards discontinuing PPI in the GERD scenario (Farrell et al., 2017). Conversely, recommendations regarding PPI gastroprotection support its usage in scenarios with moderate and high UGIB risks but not in the low-risk scenario (Bhatt et al., 2008; Abraham et al., 2010). A pilot testing of the study survey was conducted on 10 secondary and 10 primary care physicians to assess content, design, and comprehensibility. No alterations were made to the survey based on the pilot feedback.

### Data collection

The self-administered questionnaire was disseminated among secondary and primary care physicians present at their respective health facilities on the days allocated for data collection. Each health facility underwent multiple visits for data collection throughout the study duration. Participants who expressed willingness to engage in the study were provided assurances of confidentiality and were required to provide written consent before participating in the study.

### Statistical analysis

The data were entered into the Statistical Package for Social Sciences (IBM SPSS Statistics for Mac, Version 29), followed by descriptive, association, and comparative analyses. Results from the Shapiro-Wilk and Kolmogorov-Smirnov tests indicated that the data related to age, years of experience, percentage awareness and beliefs scores, and attitude scores were not normally distributed. Consequently, these results were represented as medians (interquartile ranges-IQR). Percentages were presented with 95% confidence intervals (CI). The percentage awareness score [PAS] and beliefs score [PBS] were calculated by dividing the participant’s score by 18 (the maximum score) and multiplying by 100.

The initial assessment of the association between the independent variables and outcome variables was conducted using univariate analyses. All variables displaying a significance level of *p* < 0.25 were included in the subsequent multivariable logistic regression analysis. The literature supports a cutoff value of 0.25 (Mickey and Greenland, 1989). The multivariable analyses aimed to ascertain the independent variables that are good predictors of the outcome variables. Only the results derived from the multivariable logistic analyses with significant association are reported with odds ratios (OR) and their corresponding 95% CI. Statistical significance was at *p* < 0.05. The independent variables were as follows: 1) physicians [primary care physicians, secondary care physicians]; 2). years of experience as physicians [≤9 years, >9 years]; 3) the number of patients who take PPIs they see in their practice per week [≤25, >26]; and 4) availability of decision support systems to evaluate the appropriateness for PPI continuation or discontinuation [yes, no]. The dependent variables were as follows: 1) familiarity with published scientific data on possible PPI AEs [very/somewhat, not at all/slightly]; 2) the extent of change in PPI prescribing patterns [very much/somewhat, not at all/slightly]; 3) the extent of general concern about PPIs AEs [very much/somewhat, not at all/slightly]; 4) PPI de-escalation strategies [frequently/somewhat, never/occasionally]. In addition to the above independent variables, the extent of general concern about PPI AEs [very much/somewhat, not at all/slightly] and perceived effectiveness of PPI in reducing UGIB risk in the high-risk scenario [very/moderately, Not at all/slightly] were included to identify the predictors for the appropriate management of the patient scenarios with different levels of risk for UGIB [PPI discontinuation, PPI continuation/switch to H_2_-blocker] for the minimal and low-risk scenarios, and [PPI continuation, PPI discontinuation (PPI discontinuation/switch to H_2_-blocker)] for moderate and high-risk scenarios. The Mann–Whitney test was used to evaluate the differences in the percentage awareness and belief scores between two groups of independent variables. The chi-square test was employed to compare the level of agreement on items related to attitudes toward PPI use between secondary care and primary care physicians. Statistical significance was at *p* < 0.05.

## Results

### Characteristics of the study participants

The response rate was 87.8% (541/616). Among the respondents, 53.2% were secondary care physicians. The participants’ median (IQR) age and experience as physicians were 35 (12) and 9 (11), respectively. Their familiarity with guidelines for appropriate PPI use in UGIB prevention was indicated by 461 respondents (85.2%, CI: 81.9–88.0). Additionally, 292 participants (54%, CI: 49.7–58.2) reported having decision support systems, such as guidelines or professional recommendations available to assist in determining whether PPIs should be continued or discontinued appropriately. Table 1 outlines the characteristics of the study participants.

**TABLE 1:** Characteristics of the study participants (*n* = 541).

Characteristics	*n* (%)
Physicians	
Secondary care	288 (53.2)
Primary care	253 (46.8)
Age (Years)	
≤ 35	275 (50.8)
>35	266 (49.2)
Gender	
Male	292 (54.0)
Female	249 (46.0)
Nationality	
Kuwaiti	275 (50.8)
Non-Kuwaiti	266 (49.2)
Experience as a physician (Years)	
≤ 9	271 (50.1)
>9	270 (49.9)
Patients who take PPIs seen in the practice in a typical week	
1–25	288 (53.2)
26–50	148 (27.3)
51–75	69 (12.8)
76–100	22 (4.1)
>100	14 (2.6)

### Perceptions and experiences with PPIs

Table 2 presents the perceptions and experiences of the respondents regarding PPIs. Seventy-four percent of respondents (*n* = 401, CI: 70.2–77.7) were very or somewhat familiar with published data on PPI AEs. Among the 498 respondents who indicated some level of familiarity (including slightly familiar), 346 (69.5%; CI: 65.2–73.5) stated that they had very much or somewhat modified their prescribing patterns for PPIs. Sixty-one percent of participants (*n* = 330, CI: 56.7–65.1) expressed a general concern about AEs when prescribing PPIs. Regarding communication with patients, 304 (56.2%; CI: 51.9–60.4) reported that they sometimes or often discuss the risks of AEs with patients starting PPIs. Additionally, 173 respondents (32%, CI: 28.1–36.1) indicated that patients sometimes or often express concerns regarding the PPIs AEs.

**TABLE 2:** Respondents’ perceptions and experiences with proton pump inhibitors.

	Very much *n* (%)	Somewhat *n* (%)	Slightly *n* (%)	Not at all *n* (%)
Familiar with published data on PPI AEs (*n* = 541)	127 (23.5)	274 (50.6)	97 (17.9)	43 (7.9)
Have changed PPI prescribing habits as a result of studies on PPI AEs (*n* = 498)	98 (19.7)	248 (49.8)	117 (23.5)	35 (7.0)
In general, concerned about AEs when prescribing PPIs (*n* = 541)	129 (23.8)	201 (37.2)	133 (24.6)	78 (14.4)
	Often *n* (%)	Sometimes *n* (%)	Rarely *n* (%)	Never *n* (%)
Discuss the risks of AEs with patients before starting PPI (*n* = 541)	117 (21.6)	187 (34.6)	138 (25.5)	99 (18.3)
Patients on PPI therapy bring up concerns about the risk of adverse effects from PPIs (*n* = 541)	32 (5.9)	141 (26.1)	241 (44.5)	127 (23.5)

### Awareness and beliefs about the potential AEs associated with long-term use of PPI

Table 3 shows the awareness and beliefs of respondents regarding the potential 18 AEs associated with long-term use of PPI. Respondents indicated their awareness that PPIs increase the risk for a median (IQR) of 15 (9) different AEs [Mean (SD) 12.9 (5.4)]. More than three-quarters of respondents demonstrated awareness of osteoporosis or osteopenia (90.2%), hypomagnesemia (81.5%), vitamin B12 deficiency (80.6%), bone fracture (80.0%), C. difficile infection (77.8%), calcium deficiency (77.6%), and iron deficiency (77.6%) as AEs associated with the long-term PPI use. The overall median (IQR) percentage awareness score among respondents was notably high [83.3% (50)]. Specifically, 50.5% (*n* = 273; CI: 46.2–54.8) demonstrated high awareness [PAS ≥ 80%], 19% (*n* = 103; CI: 15.9–22.7) moderate awareness [PAS = 60–79%], and 30.5% (*n* = 165; CI: 26.7–34.6) low awareness [PAS < 60%]. About one-third of participants (*n* = 177, 32.7%, CI: 28.8–36.9) reported awareness of the 18 specific AEs associated with the long-term use of PPIs.

**TABLE 3:** Awareness and beliefs about the potential AEs associated with long-term use of proton pump inhibitors (*in descending order according to percentage awareness*) (*n* = 541).

Adverse effect	Aware of the association with PPI use *n* (%)	Believe in the association with PPI use *n* (%)
1. Osteoporosis or Osteopenia	488 (90.2)	439 (81.1)
2. Hypomagnesemia	441 (81.5)	363 (67.1)
3. Vitamin B12 deficiency	436 (80.6)	337 (62.3)
4. Fracture of a bone	433 (80.0)	317 (58.6)
5. Clostridium difficile infection	421 (77.8)	324 (59.9)
6. Calcium deficiency	420 (77.6)	290 (53.6)
7. Iron deficiency	420 (77.6)	293 (54.2)
8. Chronic Kidney Disease	402 (74.3)	229 (42.3)
9. Gastric cancer	396 (73.2)	198 (36.6)
10. Acute Interstitial Nephritis	391 (72.3)	279 (51.6)
11. Pneumonia	377 (69.7)	181 (36.2)
12. Dementia	359 (66.4)	121 (22.4)
13. Nontyphoid Salmonella and Campylobacter infections	343 (63.4)	118 (21.8)
14. Hepatic encephalopathy	338 (62.5)	104 (19.2)
15. Stroke	335 (61.9)	99 (18.3)
16. Hepatocellular carcinoma	332 (61.4)	73 (13.5)
17. Death due to cardiovascular disease, chronic kidney disease, and upper GIT cancer	332 (61.4)	108 (20.0)
18. Myocardial infarction	327 (60.4)	85 (15.7)

Respondents endorsed believing that PPIs elevate the risk for a median (IQR) of 7 (6) [Mean (SD) 7.3 (4)] different AEs, most often osteoporosis or osteopenia (81.8%), followed by hypomagnesemia (67.1%), vitamin B12 deficiency (62.3%), C. difficile infection (59.9%), and bone fracture (58.6%). The overall median (IQR) percentage beliefs score among respondents was notably weak [38.9% (33.3)]. Specifically, 80.2% (*n* = 434; CI: 76.6–83.5) demonstrated weak beliefs [PBS < 60%], 14.3% (*n* = 77; CI: 11.5–17.5) moderate beliefs [PBS = 60%–79%], and 5.6% (*n* = 30; CI: 3.8–7.9) strong beliefs [PBS ≥ 80%]. The AEs that respondents expressed the greatest concern about in clinical settings when prescribing PPIs were osteoporosis or osteopenia (32.7%), followed by acute interstitial nephritis (10.7%) and *C. difficile* infection (9.6%).

### Changes in PPI prescribing practices due to concerns regarding PPI-related AEs

Table 4 displays the strategies employed by respondents to de-escalate PPI usage due to concerns about the potential harms associated with long-term PPI therapy. Over half of respondents sometimes or frequently recommend simply stopping the PPI (*n* = 330, 61%, CI: 56.7–65.1) and using PPI only on-demand/as-needed (*n* = 313, 57.9%, CI: 53.6–62.0).

**TABLE 4:** Strategies for proton pump inhibitors de-escalation *(in descending order according to percentage frequently/sometimes)* (*n* = 541).

Strategy	Frequently *n* (%)	Sometimes *n* (%)	Occasionally *n* (%)	Never *n* (%)
Simply stop the PPI	123 (22.7)	207 (38.3)	132 (24.4)	79 (14.6)
Recommend using PPI only on-demand/as needed instead of daily	166 (30.7)	147 (27.2)	101 (18.6)	127 (23.5)
Reduce daily PPI dose from a standard dose to half of a standard dose (e.g., omeprazole 10 mg daily)	67 (12.4)	162 (29.9)	143 (26.4)	169 (31.2)
Slowly taper a daily PPI	58 (10.7)	163 (30.1)	127 (23.5)	193 (35.7)
Stop daily PPI, and prescribe an H2-blocker (e.g., Nizatidine or Famotidine) for the first few weeks after discontinuation to prevent rebound symptoms	24 (4.4)	158 (29.2)	97 (17.9)	262 (48.4)
Substitute daily PPI with a daily H2-blocker (e.g., Nizatidine or Famotidine)	28 (5.2)	146 (27.0)	118 (21.8)	249 (46.0)

### Attitudes toward PPI use

A substantial majority (*n* = 473, 87.4%, CI: 84.3–90.1) expressed agreement or strong agreement that the overuse of PPI is commonly present in Kuwait. This was closely followed by the necessity to carry out large-scale education on the rational use of PPI for both medical staff and the general public (*n* = 423, 78.2%, CI: 74.4–81.6), Furthermore, a significant portion indicated that using PPI for a short duration does not cause significant adverse reactions (*n* = 384, 71.0%, CI: 66.9–74.7), while a considerable number foresaw that overuse of PPI will cause an increase in adverse drug reactions and medical costs (*n* = 375, 69.3%, CI: 65.2–73.1). Among the respondents, a majority agreed or strongly agreed that the main cause of PPI overuse is patients’ abuse of PPI (*n* = 345, 63.8%, CI: 59.5–67.8), and the main purpose of PPI overuse is stress ulcer prophylaxis (*n* = 321, 59.3%, CI: 55.1–63.5). There was also notable agreement that over-the-counter dispensing of PPIs from the community pharmacy should be restricted (*n* = 296, 54.7%, CI: 50.4–59.0) and that the main cause of PPI overuse is doctors’ abuse of PPI (*n* = 292, 54.0%, CI: 49.7–58.2). Primary care physicians demonstrated a significantly higher level of agreement than their secondary care counterparts in five items. Conversely, secondary care physicians expressed a significantly higher level of agreement than primary care counterparts in two items. However, no significant difference was observed between both groups in terms of agreement regarding the item “overuse of PPI is commonly present in Kuwait”. Table 5 outlines the respondents' attitudes towards the PPIs use.

**TABLE 5:** Attitudes toward the proton pump inhibitors use (*in descending order according to percentage agreed/strongly agreed*) (*n* = 541).

	Strongly Agreed/Agreed *n* (%)	Neutral *n* (%)	Strongly disagreed/Disagreed *n* (%)	Median^a^ (IQR)	Strongly Agreed/Agreed *n* (%) Physicians		p-value
					Secondary care [*n* = 288]	Primary care [*n* = 253]	
Overuse of PPI is commonly present in Kuwait	473 (87.4)	58 (10.7)	10 (1.9)	5 (1)	246 (85.4)	227 (89.7)	0.132
Necessary to carry out large-scale education on the rational use of PPI for both medical staff and the general public	423 (78.2)	101 (18.7)	17 (3.1)	4 (1)	199 (69.1)	224 (88.5)	<0.001
The use of PPI for a short duration does not cause significant adverse reactions	384 (71.0)	120 (22.2)	37 (6.8)	4 (1)	182 (63.2)	202 (79.8)	<0.001
Overuse of PPI will cause an increase in adverse drug reactions and medical cost	375 (69.3)	143 (26.4)	23 (4.3)	4 (2)	179 (62.2)	196 (77.5)	<0.001
The main cause of PPI overuse is patients’ abuse of PPI	345 (63.8)	135 (25.0)	61 (11.3)	4 (2)	155 (53.8)	190 (75.1)	<0.001
The main purpose of PPI overuse is Stress ulcer prophylaxis	321 (59.3)	129 (23.8)	91 (16.8)	4 (1)	204 (70.8)	117 (46.2)	<0.001
Over-the-counter dispensing of PPIs from the community pharmacy should be restricted	296 (54.7)	166 (30.7)	79 (14.6)	4 (1)	130 (45.1)	166 (65.6)	<0.001
The main cause of PPI overuse is doctors’ abuse of PPI	292 (54.0)	148 (27.4)	101 (18.6)	4 (2)	177 (61.5)	115 (45.5)	<0.001

^a^ Responses rated on a Likert scale ranging from 1 = strongly disagree to 5 = strongly agree.

### Management of the clinical scenario with varying levels of UGIB

Recommendations for PPI discontinuation should prioritize scenarios related to GERD, with 43.6% (CI: 39.4–47.9) of participants advocating for discontinuation in this minimal-risk situation. Similarly, in the low-risk scenario, the recommendation for PPI discontinuation was supported by 33.6% (CI: 29.7–37.8). Conversely, for gastroprotection, the consensus leaned towards PPI continuation in moderate-risk and high-risk scenarios for preventing UGIB. Specifically, 33.1% (CI: 29.2–37.3) recommended PPI continuation in the moderate-risk UGIB scenario, while a majority of 56.0% (CI: 51.7–60.2) endorsed PPI continuation in the high-risk UGIB prevention scenario. In the high-risk scenario, opinions on the effectiveness of PPIs for preventing UGIB varied, with 2.4% considering them not effective at all, 21.1% perceiving them as slightly effective, 39.0% endorsing moderate effectiveness, and 37.5% deeming them very effective. Table 6 displays the respondents’ recommendations for the management of PPI in clinical scenarios with varying levels of risk for UGIB.

**TABLE 6:** Respondents’ recommendations for the management of proton pump inhibitors in clinical scenarios with varying levels of risk for UGIB (*n* = 541).

Clinical scenarios including varying levels of UGIB risk	Recommendations for the management of PPI		
	Continue PPI *n* (%)	Discontinue PPI *n* (%)	Switch to H_2_-blocker *n* (%)
Minimal risk (GERD)	78 (14.4)	236 (43.6)	227 (42.0)
Low risk	198 (36.6)	182 (33.6)	161 (29.8)
Moderate risk	179 (33.1)	201 (37.2)	161 (29.8)
High risk	303 (56.0)	113 (20.9)	125 (23.1)

### Predictors influencing the outcome variables

#### Physicians

The primary care physicians, in comparison to their secondary counterparts, exhibited a significantly notable prevalence in several aspects: a change in prescribing practices due to recent studies about AEs of PPIs (OR: 1.7, CI: 1.1–2.5, *p* = 0.01), higher concerns about AEs during PPI prescribing (OR: 2.1, CI: 1.5–3.1, *p* < 0.001), and more frequent recommendations for PPI discontinuation in the low-risk scenario (OR: 2.9, CI: 1.9–4.4, *p* < 0.001).

In contrast, secondary care physicians demonstrated significantly greater awareness of the 18 AEs related to long-term PPI use (Mean rank: 308.2 vs. 228.6, *p* < 0.01), more common recommendations for PPI de-escalation strategy of PPI discontinuation (OR: 1.6, CI: 1.1–2.3, *p* = 0.013), and a greater tendency to recommend PPI continuation in both moderate- and high-risk UGIB prevention scenarios (OR: 3.4, CI: 2.2–5.3, *p* < 0.001) and (OR: 3.7, CI: 2.4–5.8, *p* < 0.001), respectively.

#### Experience as physicians

Physicians with experience >9 years compared to those with ≤9 years, demonstrated significantly higher levels of familiarity with published data on PPI AEs (OR: 1.8, CI: 1.0–3.1, *p* = 0.04) and were more likely to change prescribing practices due to recent studies about PPIs AEs (OR: 1.9, CI: 1.2–3.2, *p* = 0.04). Additionally, they more frequently recommended PPI de-escalation strategies, including dose reduction (OR: 1.9, CI: 1.2–3.1, *p* = 009) and gradual tapering (OR: 2.6, CI: 1.6–4.3, *p* < 0.001).They were more inclined to discontinue PPIs for minimal UGIB risk prevention (OR: 2.3, CI: 1.5–3.3, *p* < 0.001) and less likely to continue PPI use in high-risk scenarios (OR: 0.6, CI: 0.4–0.8, *p* = 0.006).

#### Decision support systems in practice for appropriate PPI use

Physicians with decision support systems for appropriate PPI use, compared to those without, demonstrated significantly higher familiarity with published data on PPI AEs (OR: 1.7, CI: 1.2–2.6, *p* = 0.006) and were more likely to change their prescribing practices due to recent studies about AEs (OR: 2.4, CI: 1.6–3.4, *p* < 0.001), and exhibited higher concerns about AEs during PPI prescribing (OR: 1.8, CI: 1.2–2.5, *p* = 0.003). They were also more inclined to recommend various PPI de-escalation strategies (*p* < 0.05).

#### PPI-taking patients per week seen by physicians in their practice

Physicians who see 26 or more PPI-taking patients per week, in comparison to those seeing 25 or fewer, were more likely to change their prescribing practices due to recent studies about AEs (OR: 1.7, CI: 1.1–2.5, *p* = 0.01). They also showed higher awareness (Mean rank: 292.2 vs. 252.4, *p* = 0.003) and belief in the specific AEs related to long-term PPI use (Mean rank: 302.6 vs. 243.2, *p* < 0.001). Additionally, they were also more inclined to recommend the PPI de-escalation strategy of PPI discontinuation (OR: 2.3, CI: 1.6–3.5, *p* < 0.001) and the PPI continuation for high-risk UGIB prevention scenario (OR: 1.6, CI: 1.1–2.4, *p* = 0.014). In contrast, physicians seeing 25 or fewer PPI-taking patients were more likely to recommend PPI discontinuation in minimal-risk scenario (OR: 1.6, CI: 1.1–2.3, *p* = 0.019).

#### Concern about PPI AEs and perceived PPI effectiveness in reducing UGIB risk

Respondents with higher concerns about PPI AEs were less likely to recommend continuing PPI therapy in both moderate-risk (OR: 0.4, CI: 0.3–0.7, *p* < 0.001) and high-risk (OR: 0.7, CI: 0.4–0.9, *p* = 0.038) exhibited a reduced likelihood of recommending PPI continuation in both moderate and scenarios. Conversely, they were more likely to recommend discontinuing PPI therapy in minimal risk scenario (OR: 1.6, CI: 1.1–2.3, *p* = 0.02). Notably, those who recommended continuing PPI therapy in high-risk scenario believed more strongly in its efficacy for preventing UGIB compared to those who recommended discontinuation (OR: 4.3, CI: 2.7–7.0, *p* < 0.001).

## Discussion

PPIs have been associated with an expanding array of serious AEs, yet their clinical significance remains a subject of ongoing debate. Against the backdrop of this uncertainty, there is a dearth of understanding regarding physicians’ awareness, beliefs, and concerns about these potential AEs and the impact of these on their prescribing practices. To the best of our knowledge, this study represents a pioneering effort in Kuwait and only the third of its kind in the MENA region, following similar investigations conducted in Saudi Arabia (Asdaq et al., 2021) and Syria (Swed et al., 2023). However, in comparison to the study in Saudi Arabia, our research offers more comprehensive insights into physicians' perceptions and experiences regarding PPIs, their awareness and beliefs regarding all published AEs associated with PPIs, alterations in prescribing practices prompted by these AEs, and their recommendations concerning the continuation or discontinuation of PPI usage in scenarios involving varying degrees of risk for UGIB. Additionally, this study marks the fourth of its kind globally, succeeding two analogous investigations conducted in the USA (Kurlander et al., 2018; Kurlander et al., 2020) and one in Syria (Swed et al., 2023). Significantly, our research distinguishes itself by elucidating physicians' awareness and beliefs regarding 18 specific AEs, surpassing the coverage of 12 specific AEs examined in recent studies conducted in the USA (Kurlander et al., 2020) and Syria (Swed et al., 2023).

In the present study, 74.1% of participants were somewhat or very familiar with published data on PPI AEs, and 69.5% acknowledged that they had somewhat or very much changed their practices in prescribing PPIs due to recent studies on AEs. These percentages are comparatively lower than those reported in the USA 93% and 76%, respectively, by Kurlander et al. (2020), yet higher than those documented in Syria 52.5% and 64.3%, respectively, by Swed et al. (2023). Furthermore, it is noteworthy that 61% of respondents expressed concern about AEs associated with PPIs, which is relatively lower compared to the 70% reported in the USA by Kurlander et al. (2020), yet higher than the 32.7% documented in Syria by Swed et al. (2023). The present findings elucidate that respondents exhibited familiarity with the reported PPI AEs, expressed concerns regarding AEs when prescribing PPIs, and changed their PPI prescribing behavior notwithstanding the ongoing debate regarding the true risks posed by PPIs. The discerned patterns in these findings unequivocally underscore the manifestation of a noteworthy level of apprehension among participants, indicating that the published potential AEs linked to PPIs is giving rise to a measure of alarm among physicians in Kuwait.

More than three-quarters of respondents demonstrated awareness of seven AEs associated with long-term PPI usage including osteoporosis or osteopenia, followed by hypomagnesemia, vitamin B12 deficiency, bone fracture, C. difficile infection, calcium deficiency, and iron deficiency. These findings diverge from those reported in the USA, where ≥75% of physicians were aware of osteoporosis or osteopenia/bone fracture, followed by C. difficile infection, pneumonia, and vitamin B12 deficiency (Kurlander et al., 2020). Despite the absence of conclusive evidence establishing a causal relationship between PPIs and many associated AEs, respondents endorsed believing that PPIs increase the risk for a mean (SD) of 7.3 (4) different AEs. Notably, the AEs perceived as most likely to be associated with PPIs included osteoporosis or osteopenia (81.8%), followed by hypomagnesemia (67.1%), vitamin B12 deficiency (62.3%), C. difficile infection (59.9%), and bone fracture (58.6%). This stands in contrast to findings from studies conducted in the USA and Syria (Kurlander et al., 2020; Swed et al., 2023). In the USA, physicians believed that PPIs increased the risk for a mean (SD) of 5.2 (2.5) different AEs, with the most commonly endorsed AEs being osteoporosis or osteopenia/bone fracture (88%), C. difficile infection (82%), and pneumonia (70%). In Syria, physicians believed that PPIs increased the risk for weakening of the bones (81.8%) followed by vitamin B12 deficiency (79.7%), vitamin D deficiency (79.5%), and osteoporosis or osteopenia/bone fracture (77.6%). Moreover, in our study, the AEs for which respondents expressed the greatest concern in clinical settings when prescribing PPIs were osteoporosis or osteopenia (32.7%) and acute interstitial nephritis (10.7%). This contrasts with physicians’ concerns in the USA, where they were most apprehensive about C. difficile infection (28%) and osteoporosis or osteopenia/bone fracture (25%), and in Syria where physicians were more concerned about dementia (99.6%) and death (98.1%). The present findings underscore the imperative for educational initiatives targeting physicians to communicate the lack of conclusive evidence establishing a causal relationship between PPIs and many AEs associated with their usage, emphasizing that the generally weak strength of association is often attributable to residual confounding factors in most cases; while the most robust evidence of a causal link is observed for enteric infections, hypomagnesemia, and fundic gland polyps (Vaezi et al., 2017), it is crucial to recognize that the scientific strength of these studies is often weak, and physicians should be educated to critically evaluate them, as many of these studies are retrospective and observational, significantly reducing their value (Savarino et al., 2016). A pivotal contribution to this discourse comes from a substantial placebo-controlled randomized trial in 2019, which reported that pantoprazole is not associated with any AEs over 3 years, except for an increased risk of enteric infections. These findings indicate that PPI therapy for a few years carries little detectable risk, while potential risks from longer-term use could not be assessed. This study concluded that PPIs should only be utilized when the anticipated benefits significantly outweigh the potential risks. Furthermore, it emphasizes the importance of adhering to recommended doses and durations of treatment (Moayyedi et al., 2019). Given the high prevalence of inappropriate PPI prescriptions in the inpatient and primary care settings (Savarino et al., 2018), it is imperative to direct educational efforts towards primary and secondary care physicians in Kuwait on the proper use of PPIs rather than solely on PPI AEs, given that awareness of PPI AEs has been widely disseminated, many of these studies have been based on less robust evidence. Therefore, enhancing education on the correct prescribing practices based on evidence-based guidelines is crucial for improving patient outcomes and reducing PPI-related AEs (Savarino et al., 2017).

Within the ongoing debate regarding the potential AEs associated with PPI, our study sheds light on the diverse strategies employed by participants to de-escalate PPI usage. Notably, the predominant approach was the PPI discontinuation (61%), followed by using PPI only on-demand/as-needed (57.9%), reducing the PPI dose (42.4%), and slowly tapering daily PPI (40.8%). This observed variability in de-escalation strategies presents a contrast to findings reported in the USA and Syria (Kurlander et al., 2020; Swed et al., 2023). In the USA, the most prevalent strategy involved reducing the PPI dose (62.6%), followed by switching to an H_2_ blocker (51.5%), and the PPI discontinuation (43.9%), while in Syria the most prevalent strategy was reducing the PPI dose (55.6%) followed by switching to an H_2_ blocker (55%), and the PPI discontinuation (36.6%). All of these approaches have been employed in PPI de-escalation studies, but none emerges as a clearly favored option based on the available evidence (Inadomi et al., 2001; Bjornsson et al., 2006; Reimer and Bytzer, 2010; Lødrup et al., 2013). It is crucial to recognize that not all PPI de-escalation strategies are universally applicable to every patient. Understanding when and how physicians make decisions regarding PPI de-escalation is a pivotal aspect of ensuring appropriate treatment. The individualized nature of risk-benefit considerations plays a pivotal role in guiding physicians to make informed decisions about the necessity of PPI therapy. Once the decision is made that a patient no longer requires PPIs, physicians must then select the most suitable de-escalation strategy. However, it is apparent that these decisions often deviate from the optimal evidence-based practices, highlighting a discordance between clinical decision-making and established best practices. Addressing this incongruity is paramount to ensuring that patients who genuinely require PPI therapy continue to receive it, while those who do not are appropriately withdrawn from such medications. The potential intervention strategy of incorporating decision support systems, such as guidelines or professional recommendations, holds promise in enhancing PPI prescribing patterns in Kuwait. This is particularly pertinent considering that 46% of participants reported a lack of decision support systems in their practice. These decision support systems can offer evidence-based insights into the optimal duration of PPI therapy, risk assessment for AEs, and appropriate strategies for de-escalation. Incorporating and adhering to decision support systems within healthcare settings can enable real-time access to evidence-based information, aiding clinicians in making informed decisions aligned with established guidelines. A prior meta-analysis has demonstrated the efficacy of the clinical decision support systems at the point of care in enhancing the prescribing practices of other recommended medications (Bright et al., 2012). However, it is imperative to meticulously assess the impact on clinician satisfaction and work efficiency, considering the widespread prevalence of PPI use.

A notable observation in the present study is the widespread acknowledgment among respondents (87.4%) that PPI overuse is prevalent in Kuwait, which is closely aligned with similar results in China (87.5%) and Saudi Arabia (92.2%) (Luo et al., 2019; Asdaq et al., 2021). This shared recognition emphasizes the global nature of concerns related to PPI usage patterns. The consensus among respondents (78.2%) regarding the imperative for large-scale education on the rational PPI use for both medical staff and the public is noteworthy and aligns with similar findings in China and Saudi Arabia. The parallel findings across diverse regions suggest a universal recognition of the challenges associated with PPI overuse and the instrumental role of education in mitigating these challenges. Implementing large-scale educational initiatives could serve as a transformative strategy, not only in promoting evidence-based PPI prescribing practices but also in empowering patients to make informed decisions about PPI usage. This aligns with a broader global trend toward promoting patient-centered care through increased health literacy. The belief among participants (71.0%) that using PPIs for a short duration does not lead to significant adverse reactions was 71% compared to 70.5% in China and 87.6% in Saudi Arabia. This reflects a shared understanding of the safety profile of short-term PPI use. However, it is crucial to consider these perceptions in light of existing evidence, which indicates that even short-term PPI use may have implications for adverse reactions (Lexicomp, 2024), thereby highlighting a potential gap between perception and evidence. Concerns about the consequences of PPI overuse, such as increased adverse drug reactions and medical costs, were expressed by 69.3% of respondents compared to 66.8% in China and 85.3% in Saudi Arabia. This indicates a common apprehension regarding the broader healthcare implications associated with excessive PPI usage. The study participants agreed or strongly agreed on multifactorial contributors to PPI overutilization. They attributed overuse to patient abuse (63.8%) and doctor abuse (54%) aligns with trends observed in China and Saudi Arabia indicating a shared perception of both contributors to PPI overutilization. Stress ulcer prophylaxis was indicated by 59.3% of participants compared to 45.1% in China and 72.9% in Saudi Arabia, emphasizing a clinical scenario where PPIs are often used without clear indications. The call for restrictions on over-the-counter dispensing of PPIs from community pharmacies was 54.7%, compared to 42% in China and 86.8% in Saudi Arabia suggesting a recognition of the role of easy access in contributing to overuse. The present results regarding the attitudes toward PPI usage among physicians in Kuwait reflect an interplay of perceptions, concerns, and proposed interventions. These insights provide a foundation for targeted educational initiatives and regulatory measures to align prescribing practices with evidence-based guidelines. Addressing these findings is crucial for optimizing patient care, minimizing adverse events, and promoting the rational use of PPIs in Kuwaiti healthcare settings.

In response to the clinical scenarios, 14.4% and 36.6% of respondents inappropriately recommended PPI continuation in minimal and low-risk UGIB prevention scenarios. This contrasts with percentages of 14% and 9% in the USA (Kurlander et al., 2020) and 16.1% and 20.3% in Syria (Swed et al., 2023), respectively. Conversely, in high-risk and moderate-risk UGIB prevention scenarios where PPI continuation is deemed appropriate, 56% and 33.1% of respondents appropriately recommended PPI continuation. This contrasts with percentages of 21% and 14% in the USA, and 23.7% and 15% in Syria, respectively. These findings suggest that respondents in our study have received the cautionary message regarding the potential harm associated with PPIs, leading to a more restrained approach in their usage when appropriately indicated. However, they also raise concerns that initiatives aimed at reducing PPI utilization may inadvertently contribute to the persisting problem of PPI use for UGIB prevention (Medlock S et al., 2013; Kurlander et al., 2019). Additionally, these results emphasize the importance for physicians to assess the risk of upper gastrointestinal complications when considering deprescribing and to understand the PPIs’ role in preventing acid-peptic diseases. A crucial finding emerged as there was no statistically significant association identified between familiarity with PPI guidelines for appropriate PPI use in the UGIB prevention or the availability of decision support systems and the appropriate recommendations for PPI discontinuation or continuation in the presented clinical scenarios (*p* > 0.05). This notable result highlights a critical consideration: the mere familiarity with guidelines or access to decision support systems may not, in isolation, lead to improved rational prescribing practices for PPIs in UGIB prevention. The absence of a clear link emphasizes the necessity for effective enforcement mechanisms within the clinical setting to translate knowledge into action. Intriguingly, our study also revealed a distinct pattern among physicians who had decision support systems, demonstrating significantly higher levels of familiarity with PPI AEs. These physicians not only altered their prescribing practices more frequently but also expressed heightened concerns about AEs during PPI prescribing. Moreover, they were more inclined to engage in discussions about AEs with their patients. This positive correlation suggests that decision support systems may play a pivotal role in enhancing physicians’ overall awareness, proactive adjustment of prescribing habits, and improved communication regarding potential risks with patients.

The study’s findings, derived from associations and comparative analysis, revealed various predictors influencing physicians’ perceptions, experiences, awareness, beliefs, practices, and attitudes regarding PPI use. Tailoring interventions considering these predictors may contribute to optimizing PPI prescribing practices and ensuring patient safety. However, further research is essential to explore the causal relationships between these predictors and outcome variables, facilitating the development of targeted interventions contributing to evidence-based practices in healthcare facilities. Primary care physicians demonstrated a notable change in prescribing practices in response to recent studies on AEs associated with PPIs. Their higher concerns about AEs during PPI prescribing, frequent discussions with patients about risks, and a proactive approach to PPI discontinuation in the low-risk UGIB scenario suggest a cautious and patient-centered prescribing behavior. On the contrary, secondary care physicians exhibited a significantly greater awareness of the increased risk of specific AEs related to long-term PPI use, showcasing a preference for the de-escalation strategy of PPI discontinuation and a tendency to recommend continuation in moderate- and high-risk scenarios. These differences underscore the need for tailored interventions and educational initiatives based on the specialty of physicians. Physicians with over 9 years of experience displayed a greater familiarity with published data on PPI AEs and were more likely to change prescribing practices in response to recent studies. Their increased adoption of de-escalation strategies may highlight the evolving nature of prescribing practices with cumulative experience. Physicians seeing 26 or more PPI-taking patients per week showed a higher frequency of changes in prescribing practices, higher awareness, and belief in the increased risk of AEs related to long-term PPI use. Their inclination towards specific de-escalation strategies and PPI continuation in high-risk scenarios could indicate the influence of patient load on prescribing decisions.

### Strengths and limitations

The strengths of this study include: i) it achieved a high response rate, underscoring the relevance and importance of the research topic among physicians; ii) its timeliness is underscored by its execution in 2023, aligning with the publication of recent studies linking PPIs to additional AEs not addressed in preceding studies; iii) it addresses a gap in the existing literature by offering insights into contemporary perspectives, awareness, and beliefs of physicians regarding scientific data on PPI AEs and their impact on prescribing practices. This contribution is particularly valuable given the limited existing literature on this specific aspect; and iv) it sets the stage for vital comparative work with current and future studies in the MENA countries and globally, fostering a broader understanding of variations and similarities in physicians’ perspectives and practices related to PPIs.

The interpretation of the present results should be approached with consideration of certain potential limitations. These include: i) its external validity with regard to the generalizability of its results to all physicians in Kuwait, which is impacted by the use of convenience sampling. The unavailability of comprehensive lists containing primary and secondary care physicians hindered the adoption of systematic random sampling. Consequently, the study relied on the participation of physicians present at healthcare facilities during the days designated for data collection, potentially introducing selection bias; ii) the responses obtained in this study are potentially influenced by social desirability bias, as participants may have chosen responses that align with socially accepted views rather than expressing their true perspectives. The reliance on self-reported data poses a challenge in verifying the accuracy of participants’ claims, and that the responses are accepted at face value. Although efforts were made to mitigate social desirability through anonymous survey completion and assurances of confidentiality, it remains a factor that should be taken into account when interpreting the findings; iii) respondents’ management recommendations in clinical scenarios are likely to mirror their perceptions of what they consider optimal management strategies. However, it is crucial to acknowledge that these recommendations may diverge from real-world clinical management. Additionally, it is worth noting that the order of the survey questions, where inquiries about perceptions of AEs preceded the scenarios, could potentially have influenced participants’ responses, and iv) the cross-sectional design employed in this study, capturing data at a single point in time, restricts the ability to infer changes over time in the responses of the study population.

## Conclusion

The present study provides a comprehensive assessment of physicians’ perceptions, experiences, awareness, beliefs, practices, and attitudes regarding PPI use in Kuwait. In relation to the specific 18 AEs associated with long-term PPI therapy, 69.6% of physicians exhibited moderate or high awareness, while 80% expressed weak beliefs. A majority of respondents expressed a general concern about AEs associated with PPIs, leading to substantial changes in prescribing patterns. The common strategies for PPI de-escalation were predominantly discontinuation and on-demand/as-needed usage. Attitudes toward PPI use reflected concerns about overuse in Kuwait and a consensus on the need for large-scale education on the rational use of PPI for both medical staff and the public. Respondents varied in their recommendations for PPI discontinuation or continuation across different UGIB prevention scenarios, 33.1% and 56% appropriately recommended PPI continuation for the moderate- and high-risk scenarios, respectively, while 43.6% and 33.6% appropriately recommended PPI discontinuation in the minimal- and low-risk scenarios. The identified predictors influencing physicians’ perceptions, experiences, awareness, beliefs, practices, and attitudes regarding PPI use could serve as signposts for tailoring targeted interventions that contribute to optimizing PPI prescribing practices and ensuring patient safety. It is crucial to focus educational efforts on primary and secondary care physicians in Kuwait regarding the proper use of PPIs, rather than solely on PPI AEs. Although awareness of PPI AEs has been widely disseminated, many of studies on AEs are based on less robust evidence. Therefore, enhancing education on the correct prescribing practices based on evidence-based guidelines is essential for improving patient outcomes and reducing PPI-related AEs.

## Data Availability

The raw data supporting the conclusion of this article will be made available by the authors, without undue reservation.
